# The gut microbiome and sociability

**DOI:** 10.3389/fnins.2024.1372274

**Published:** 2024-04-02

**Authors:** Katherine T. Weber, Bernard J. Varian, Susan E. Erdman

**Affiliations:** Division of Comparative Medicine, Massachusetts Institute of Technology, Cambridge, MA, United States

**Keywords:** *L. reuteri*, oxytocin, autism spectrum disorder, gut-brain-axis, probiotic

## Abstract

The human gut microbiome plays an important role in the maturation of the neural, immune, and endocrine systems. Research data from animal models shows that gut microbiota communicate with the host's brain in an elaborate network of signaling pathways, including the vagus nerve. Part of the microbiome's influence extends to the behavioral and social development of its host. As a social species, a human's ability to communicate with others is imperative to their survival and quality of life. Current research explores the gut microbiota's developmental influence as well as how these gut-brain pathways can be leveraged to alleviate the social symptoms associated with various neurodevelopmental and psychiatric diseases. One intriguing vein of research in animal models centers on probiotic treatment, which leads to downstream increased circulation of endogenous oxytocin, a neuropeptide hormone relevant to sociability. Further research may lead to therapeutic applications in humans, particularly in the early stages of their lives.

## 1 Introduction

The gut microbiome, made up of its thousands of species of commensal microbiota, influences the development of many facets of its host over the course of their lifetime, including the maturation of the immune system (Schachtle and Rosshart, 2021), development of the brain (Dinan and Cryan, 2017), and its host's behavior (Cryan and Dinan, 2012; Sarkar et al., 2020). As a social species, humanity's social behaviors are an important element of their survival and evolution (Wu et al., 2021), and research over the last few decades suggests the gut microbiome has influence over an individual's sociability. Uncovering the connections between gut microbiota, the brain, and sociability could lead to better understanding of psychiatric diseases and neurodevelopmental disorders that compromise sociability as well as microbial interventions that could alleviate their symptoms.

The gut microbiome communicates with the brain through intricate, bidirectional pathways of communication. One of the best-studied pathways is facilitated by the vagus nerve (Forsythe et al., 2014), which relays chemical signals between the gut and the brain, reaching the central nervous (CNS) (Bravo et al., 2011), the immune system (Belkaid and Hand, 2014), and endocrine system via the hypothalamus-pituitary-adrenal (HPA) axis (Viero et al., 2010). An important downstream target of gut-brain communication is the neuropeptide hormone oxytocin, which is paramount in an individual's ability to form social bonds (Horn and Carter, 2021) as well as an anti-inflammatory factor (Panaro et al., 2020).

Though many of these gut-brain communication mechanisms are poorly understood or undiscovered, studies suggest that an imbalance between healthy and unhealthy gut microbiota may disrupt the pathways of communication between the gut and brain and lead to aberrant phenotypes. Previous research in this field demonstrates that a dysbiotic, or unhealthy, gut microbiome is etiologically linked to neurological and psychiatric disorders such as depression, anxiety, and autism spectrum disorder (ASD) (Warner, 2019). One of the symptoms characteristic of these disorders, especially ASD, is aberrant sociability.

Research over the last decade suggests that these disorders arise early in an individual's development and can be influenced by their mother's health and gut microbiome while *in utero*, suggesting a multigenerational component to the development of these disorders (Di Gesu et al., 2022). Further, probiotic treatment, both pre- and postnatally, have been shown to alleviate gastrointestinal and sociability symptoms, like those characteristic of ASD (Abdellatif et al., 2020). Though researchers have done a lot to discover gut-brain mechanisms and effective treatments, this is a burgeoning field, and much research is left in order to discover relevant microbial interventions that can be administered early in an individual's development and how they impact the mechanisms of the gut-brain axis.

## 2 Gut microbial symbionts and sociability

In order to understand how the gut microbiome interacts with the body, researchers in this field often conduct experiments in mouse models to illuminate the role of the gut-brain-axis in orchestrating immunomodulatory outcomes. Outcomes highlight the apparent symbiotic nature of the relationship between a host and their resident microbiota. Revealing these mechanisms provides a foundation for understanding the etiology of these neurological and psychiatric disorders. One of the best understood communication pathways between the gut and brain is the vagus nerve. A series of experiments conducted by Poutahidis et al. (2013a) involved vagotomies and probiotic treatment in a wound healing mouse model system to investigate the role of the vagus nerve in gut-brain communication in healthful phenotypes (Levkovich et al., 2013). These studies used a probiotic *Lactobacillus reuteri* (ATC-PTA-6475) derived from human breast milk (Poutahidis et al., 2013a), that was since reclassified as the *Limosilactobacillus* family (Zheng et al., 2020). Animals with an intact vagus nerve that were dosed with *L. reuteri* had accelerated wound healing rates; animals that were treated and received a vagotomy did not experience improvements in wound healing capacity (Poutahidis et al., 2013a). Previous research established the health benefits of *L. reuteri* and Poutahidis et al. (2013a) builds on this body of research. However, there are many other candidate probiotics that relay health benefits to their host and contribute to gut-brain communication. Other species of *Limosilactobacillus* have similar health benefits to *L. reuteri*. *Lactobacillus rhamnosus* specifically has been shown to reduce inflammation in asthma models (Spacova et al., 2020). *Bifidobacterium infantis* is another important colonizer of infant guts (Batta et al., 2023) and modulates inflammatory response similar to *L. reuteri* (Groeger et al., 2013).

Results from this study also demonstrated that the breast milk-borne microbe stimulated an increase in hypothalamic hormone oxytocin (Poutahidis et al., 2013a), a hormone associated with sociability and an anti-inflammatory factor that helps keep an individual healthy (Panaro et al., 2020). This suggests a link between gut health and the host's health. Varian et al. (2017) showed microbiota and their factors stimulate a reciprocal relationship between oxytocin and stress hormone corticosterone in mice. This data supports other research which asserts that a healthy gut microbiome attenuates the body's stress hormones, specifically cortisol in humans and corticosterone in rodents. The HPA axis, which is in part influenced by communication via the gut-brain axis (Viero et al., 2010), releases and circulates these stress hormones in the body (Sudo et al., 2004; Appleton, 2018). Maintaining a healthy gut microbiome is imperative to maintaining balance in the host's immune and stress responses. An unhealthy gut microbiome can disrupt these important pathways that confer health benefits to the host. This often occurs when there is an imbalance of beneficial microbial taxa like *Bacteroides* and harmful microbial taxa like *Clostridium* (Abuaish et al., 2021). The gut-brain axis cannot send signals throughout the body with a dysbiotic gut environment as effectively has a healthy gut microbiome (Schachtle and Rosshart, 2021). Imbalanced gut microbiomes are associated with many inflammatory diseases like IBS, cardiovascular diseases, and obesity (Taniya et al., 2022).

A series of pioneering experiments fed mice a high-fat and low fiber, or “Western” diet to see how a poor diet altered the landscape of the gut microbiome and how it impacted the host's health (Poutahidis et al., 2013b). Their analysis found that the Western diet restructured the landscape of the animals' gut microbiomes, ultimately leading to obesity (Poutahidis et al., 2013b). Notably, these mice also showed markers of heightened and prolonged inflammation and more crown-like structures in their abdominal fat. Rampant stress and inflammation instigated by an unbalanced gut microbiome may lead to downstream negative effects on social development and behaviors (Peirce and Alvina, 2019; Pearson-Leary et al., 2020).

## 3 Multigenerational implications of gut microbiome

**Figure 1:**
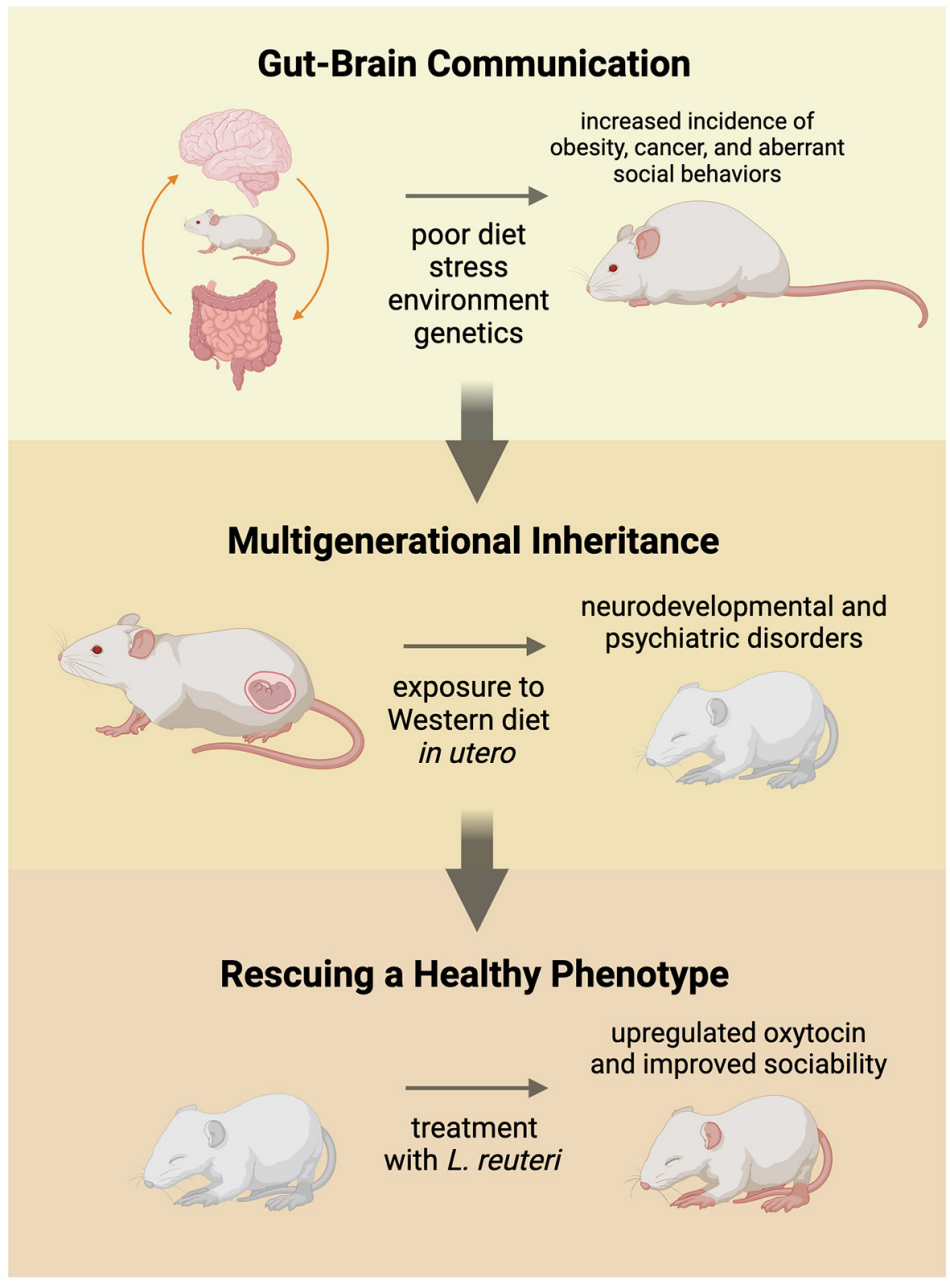
Commensal gut microbiota communicate with the brain to influence the social development and health of the individual. A balanced gut microbiome maintains homeostasis in the body and keeps the host healthy. When fed a high-fat, low-fiber (Western) diet over an extended period of time, the gut microbiome becomes unbalanced and can lead to increased incidence of obesity, cancer, and stunted sociability. Pregnant animals fed a Western diet experienced higher levels of stress and passed on their unhealthy gut microbiome to their offspring *in utero*. Weanlings experienced increased prevalence of cancer, obesity, and neurodevelopmental and psychiatric disorders, including aberrant social behaviors. Offspring that were treated with probiotics, like *Lactobacillus reuteri*, experienced alleviated symptoms, lower levels of obesity and cancer, and improved sociability.

In an attempt to uncover the origin of social abnormalities associated with an imbalanced gut microbiome, researchers have turned to the pre- and postnatal environment (Figure 1). One thread of research has established a link between maternal immune activation (MIA) and neurological disorders (Han et al., 2021). MIA may be caused by stress, chronic inflammation, infection, and a poor diet, which disrupts the balance of the gut microbiome and increases the pro-inflammatory immune response in the body (Han et al., 2021). Exposure *in utero* to maternal MIA increases an individual's risk to develop ASD, as well as other psychiatric and neurological disorders, like schizophrenia and bipolar disorder (Conway and Brown, 2019; Bordeleau et al., 2020). This coincides with the link between maternal obesity, which is an inflammatory disorder, and ASD diagnosis (Li et al., 2016).

In the high-fat, low-fiber diet experiments, offspring of pregnant dams that were fed the Western diet *in utero* experienced higher risks for obesity, infertility, premature aging, and cancer, suggesting multigenerational health consequences of the diet (Poutahidis et al., 2015). Mice in these studies responded favorably to treatment of purified *L. reuteri*, supporting an underlying microbial mechanism. In a recent study where mice were exposed to a high-fat diet *in utero*, the mice saw improvements after being treated with *L. reuteri* as well (Di Gesu et al., 2022). The researchers assert that the *L. reuteri* increased the abundance of beneficial short-chain fatty acids (SCFAs), which may be responsible in-part for improving the symptoms of the affected offspring mice (Di Gesu et al., 2022). Another potential method of treatment was conducted by Poutahidis et al. (2015). They performed fecal microbiota transplants of the dysbiotic microbes to germ-free dams (Poutahidis et al., 2015) to demonstrate a causal link between Western diet, microbiota and multigenerational health outcomes (Figure 1). Buffington et al. (2016) later validated and expanded on this phenomenon; the offspring of mothers fed a high-fat diet displayed impaired social behaviors and tended to avoid novel social experiences. The social behaviors seen in these mice is akin to the social tendencies observed in humans with autism spectrum disorder (ASD). Aberrant social behavior is a common symptom in individual's diagnosed with ASD, as well as other neurological and psychiatric disorders (Vuong and Hsiao, 2017). Sgritta et al. (2019) used a *Shank*^−/−^ mouse model that exhibits social behaviors similar to those of individuals with ASD. These animals, like those fed the Western diet, presented altered gut microbial ecosystems, including reduced levels of *L. reuteri* compared to control wild-type littermates (Sgritta et al., 2019).

Many individuals diagnosed with ASD experience gastrointestinal comorbidities, like diarrhea and constipation, along with the characteristic sociability abnormalities (Kang et al., 2017). Similar to the *Shank*^−/−^ mice, analysis of the gut microbiota of individuals with ASD have revealed that many experience gut dysbiosis partially attributed to a decrease in beneficial bacterial taxa, like *Bacteroides* and *Firmicutes* (Vuong and Hsiao, 2017). This pattern implies a link between the resulting gut dysbiosis and their accompanying social behaviors similar to what is seen in animal models (Frye, 2018). Comparable observations have been made in various models of other psychiatric and neural disorders, including animal models for anxiety and depression (Peirce and Alvina, 2019; Pu et al., 2021), unmedicated schizophrenia patients (Zhu et al., 2020), and recently a potentially microbially-induced mouse model of fragile X syndrome (Al Olaby et al., 2022; Varian et al., 2022). A complete lack of gut microbiota diversity can also have harmful effects on the health of the host. Germ-free mice exhibit decreased sociability and are less likely to partake in novel social experiences (Crumeyrolle-Arias et al., 2014; Vuong et al., 2017). More common in humans, an aggressive antibiotic treatment can greatly reduce diversity and extent of an individual's microbial flora, disrupting the brain-gut signaling pathways and negatively altering social behaviors (Desbonnet et al., 2015; Johnson and Burnet, 2020).

By leveraging this established link between the gut, the brain, and the immune system, treatment focused upon the gut microbiome may alleviate these symptoms of poor sociability. Indeed, researchers have tested probiotic interventions that apparently restore balance in the microbial environment. In multigenerational studies in animal models, it was discovered that the offspring experienced improved good health from *L. reuteri* supplementation in drinking water (Ibrahim et al., 2014; Poutahidis et al., 2015; Varian et al., 2022). According to results from Buffington et al. (2016) and Sgritta et al. (2019), part of this healthy phenotype includes the improvement in social behaviors. While these studies have validated the potential therapeutic use of intact live microbial treatment, lysates of *L. reuteri* alone were shown to be sufficient to treat dysbiotic gut microbiomes and recover a healthy phenotype (Varian et al., 2017). Though these interventions have been largely tested on animal models, when considering future therapies viable for humans, treatment with a lysate avoids the possibility of the microbe entering the bloodstream and causing further complications. Fecal and microbial transplants from people with healthy guts to individuals with dysbiotic guts have effectively reduced sociability symptoms, setting the stage for potential long-term treatments in the future as well (Kang et al., 2017; Pu et al., 2021).

## 4 Upregulation of oxytocin and treatment implications

One of the most important parts of this field of research focused on understanding how the gut microbiome influences humans is finding interventions and therapies that work and determining when to administer them to yield the best results. Many of the previously mentioned studies investigated the downstream effects of *L. reuteri* supplementation, and a notable finding is that *L. reuteri* upregulates oxytocin, a neuropeptide hormone produced in the hypothalamus initially discovered due to its importance in pregnancy (Camerino, 2023). Its release stimulates contractions, lactation, and bonding between newborn and mother which led to its colloquial name, the “love hormone” (Magon and Kalra, 2011). An individual's ability to develop social bonds throughout their lives and even find a potential life partner is profoundly influenced by oxytocin circulation and abundance in the body, and some researchers even hypothesize that oxytocin is a driving force human evolution considering humanity's reliance on sociability (Horn and Carter, 2021). Without oxytocin, individuals are prone to be less sociable and mothers are more likely to be neglectful (Lach et al., 2018), as is the case in studies where mice do not express normal oxytocin levels due to a genetic deletion of the oxytocin receptor gene (Winslow and Insel, 2002; Lerer et al., 2008).

Current research in this field hypothesizes and studies the increase of oxytocin in the body brought on by probiotics as an outcome of gut-brain communication via the HPA axis (Poutahidis et al., 2013a). The mice studied by Poutahidis et al. (2013a) showed increased endogenous oxytocin levels alongside a rescue of a healthy phenotype after being treated with *L. reuteri*. In other studies where mice were treated with *L. reuteri*, the increased oxytocin levels correlated with improvement in the mouse's social deficits (Buffington et al., 2016; Sgritta et al., 2019) (Figure 1). A recent study also found that intestinal epithelia secrete oxytocin after the host is treated with *L. reuteri* (Danhof et al., 2023), which may be a possible future avenue of research for understanding why specifically *L. reuteri* is so effective at rebalancing the gut microbiome. Further down the signaling cascade, researchers hypothesize that oxytocin fluctuates GABA-mediated mechanisms in the brain, balancing inhibitory and excitatory factors (Lopatina et al., 2018), thought the extent of its abilities and the signaling pathway it uses to accomplish this are still unknown. More recent research demonstrated that microbiota composition and sex are both factors that influence oxytocin binding rates in the body (Effah et al., 2021), though a lot is still left to learn. Another avenue of research is analyzing the efficacy of treatment with exogenous oxytocin, which has achieved similar rescued phenotypes in animal models. While exogenous oxytocin has not been widely tested in humans for this purpose, exogenous oxytocin administration is used to stimulate contractions in women during labor and is currently being tested in individuals with ASD as a potential treatment (Miller et al., 2017; Huang et al., 2021).

## 5 Discussion

Further research must be done in order to better understand the mechanisms that coordinate the body's gut microbial, brain, immune, and endocrine response to environmental and genetic stimulus. Validating microbial therapies in animal models and beginning clinical trials in humans will yield helpful data concerning the efficacy of probiotics as a useful biomedical intervention. There is still much to be discovered about how probiotics like *L. reuteri* interact with a host's gut microbiome, how they relay signals to the brain, and the cascade of mechanisms that lead to downstream Effects we see like immune factor modulation, oxytocin regulation, and inflammatory disease development or prevention. There are many neurodevelopmental and psychiatric disorders that could benefit from gut microbial interventions, and what we learn from disorders like ASD can be applied to other conditions in the future.

## Author contributions

KW: Writing – original draft, Writing – review & editing. BV: Writing – original draft, Writing – review & editing. SE: Conceptualization, Formal analysis, Funding acquisition, Project administration, Resources, Supervision, Writing – original draft, Writing – review & editing.
